# Just Checking It Out? Motivations for and Behavioral Associations With Visiting “Slutpages” in the United States and Australia

**DOI:** 10.3389/fpsyg.2021.671986

**Published:** 2021-06-25

**Authors:** Elizabeth M. Clancy, Megan K. Maas, Evita March, Dominika Howard, Bianca Klettke

**Affiliations:** ^1^School of Psychology, Deakin University, Burwood East, VIC, Australia; ^2^Human Development and Family Studies, Michigan State University, East Lansing, MI, United States; ^3^School of Science, Psychology, and Sport, Federation University Australia, Berwick, VIC, Australia

**Keywords:** sexting, pornography, gender, slutpages, image-based sexual abuse, sexual harassment

## Abstract

“Slutpages” are a pernicious form of online image-based evaluative voyeurism (OIBEV), whereby (sexualized) images of women are posted on webpages for (predominantly) male groups to rate and comment. Despite media and public concern, OIBEV sites have garnered limited empirical study. This paper presents the first analysis of OIBEV site visitation motivations across United States and Australian samples. Participants comprised a convenience sample of 1148 young adults aged 18 to 29 years (*M* = 22.54, *SD* = 2.50); 53.0% women, 47.0% men; 54% residing in the U.S. and 46% in Australia. Respondents completed an online questionnaire. Overall, 23% of United States and 16% of Australian respondents had visited OIBEV sites. OIBEV site visitation was uniquely associated with gender and country (with men and United States being more likely to visit OIBEV sites), requesting and disseminating sexts and having one’s own image shared. Cyberbullying perpetration was associated with reduced odds of OIBEV site visitation. Motivations differed by gender, with men (80%) being most likely to visit sites to “check them out” while women were equally likely to check it out (41%) or to see if they were depicted (36%). For men, unique predictors of OIBEV site visitation were having requested, disseminated and received disseminated sexts, lower levels of anxiety and reduced likelihood of cyberbullying perpetration. For women, OIBEV site visitation was uniquely associated with being a United States resident, sext dissemination victimization, receipt of disseminated sexts, higher levels of anxiety but reduced stress. Our findings confirm that OIBEV sites represent a highly gendered form of online image-based sexual abuse, and may have important mental health implications, given the associations with increased anxiety. Our results support the need for “slutpage” education for adolescents and young adults to address social and peer norms that encourage and support non-consensual use of intimate images.

## Introduction

Emerging adults in Western society spend increasing time online communicating via messenger apps, social media, and blogs. For example, usage among United States and United Kingdom emerging adults has been estimated at over 3 h per day, in addition to work and/or study requirements (Code Computer Love, 2019; Mackay, 2019). Although some aspects of this communication are positive and adaptive, online communication and social media can also negatively impact functioning and overall well-being. For example, the ease and scope with which users can create and share sensitive or harmful information and media has been linked to poorer mental health outcomes for affected individuals (Best et al., 2014; Keles et al., 2020). These harmful forms of communication include the non-consensual sharing of sexual content, particularly explicit images of individuals, conceptualized as image-based sexual abuse (Powell et al., 2018; Henry and Flynn, 2019). Although not all non-consensual sharing of explicit information is motivated by malicious intentions (Clancy et al., 2019), such behaviors can be aimed to publicly shame, threaten, or abuse those depicted (McGlynn et al., 2017). In this paper, we focus on the under-researched phenomen of “slutpages,” a specific form of image-based sexual abuse that is more secretive, yet social.

The term “slutpages” refers to digitally created groups, websites, or email listservs where individuals share sexually explicit images of others, most often girls and women, usually without their knowledge or consent (Sales, 2016, 2017). Others, usually peers and not necessarily public audiences, are invited to view and comment on the appearance, sexuality, and/or sexual performance of those depicted (Uhl et al., 2018). It is important to acknowledge that the term “slutpages” is itself problematic, as it attributes blame and negative connotations toward those depicted. To avoid these concerns, we have chosen to use a less value-laden term, referring to online image-based evaluative voyeurism (OIBEV), but acknowledge the term “slutpages” is perhaps a more commonly known term in general discourse.

Specific instances of OIBEV have been identified in schools, college-based groups, and the military (Sales, 2016, 2017). In 2014, a United States University fraternity was suspended for creating a private Facebook group, where explicit photos of intoxicated, and in some cases allegedly unconscious, female students were shared (Garrity and Blinder, 2015). In Australia, OIBEV behaviors have been reported predominantly in elite private school settings; whereby male students post photos of female peers with the opportunity for others to comment and assess or evaluate these women in relation to their appearance, attractiveness, or other criteria (Cook, 2016; Houston and Cook, 2016; Olding, 2016; Carmody, 2017). Thus, the creation of, and visitations to, OIBEV sites is not unique to the United States.

The images that are uploaded to these OIBEV sites are often obtained via sexting (Sales, 2016, 2017), a form of sexual digital communication encompassing sending, receiving, or forwarding of sexually explicit messages, images, or photos to others through electronic means (Klettke et al., 2014). However, many of the women whose images have been shared on OIBEV sites report being unaware of the existence of these sexually explicit pictures and/or the OIBEV sites (e.g., Garrity and Blinder, 2015). This implies that some, if not most, of the images of such sites may have been taken without the knowledge or consent of the victimized person(s), whether due to intoxication (Garrity and Blinder, 2015), or other voyeuristic behaviors, such as upskirting or downblousing. Effects on those who become aware of being victimized on such sites are significant, with reports of increased anxiety and suicidal thoughts (Sales, 2016), and fear for one’s physical safety (Sales, 2017). Given the potential for such negative impacts for victims, it is concerning that research regarding this behavior remains in its infancy.

To date, there has been minimal empirical research addressing OIBEV. In a United States -based college sample, Maas et al. (2021) found that 33.7% of emerging adults reported having visited “slutpages,” whilst 2.8% had posted images to such sites. Men (6.5%) were significantly more likely to post images than women (0.8%). Younger respondents were more likely than older participants to both visit and post on such sites, particularly if they were engaged in Greek Life (fraternities and sororities) or participated in team sports. Alcohol and pornography use were also associated with increases in both viewing and posting to OIBEV sites. In another study exploring OIBEV, albeit obliquely, Walker et al. (2019) found that 2% of adults reported having non-consensually posted images publicly on social networking sites. However, this study was limited by (1) the lack of investigation of motivations for such behaviors, and (2) the focus on general social networking sites, instead of specifically exploring closed or secret webpages such as OIBEV sites. A third qualitative study from Italy explored the non-consensual diffusion of intimate images via Telegram, an app which facilitates large groups or channels whilst maintaining anonymity (Semenzin and Bainotti, 2020). This study noted that such large, and usually male, online communities support and facilitate online image-based harassment, and can reinforce norms of hegemonic masculinity. Despite these emerging studies, motivations for posting to, visiting, and viewing OIBEV sites are yet to be clarified.

Conceptually, OIBEV can be likened to other forms of image-based sexual abuse (McGlynn et al., 2017; Powell et al., 2018). As with other forms of sexual violence, image-based sexual abuse is considered a gendered issue, with younger women and sexual minorities over-represented as victims (Henry and Powell, 2018). Although research investigating this space is limited, such differences are unsurprising. Younger women (Kussin-Shoptaw et al., 2017) and sexual minorities (Mellgren et al., 2018) experience high rates of sexual violence offline. Such behaviors may be attributed to *cis*-gendered and heteronormative norms, attitudes about gender and sexuality, power-related motivations for perpetration, and victim blaming attitudes (Henry and Powell, 2018).

Online image-based evaluative voyeurism sites, which are generally created and shared amongst male peer groups, may also be attributed to similar *cis*-gendered, heteronormative norms and attitudes about sexuality, gender and roles, as they can form bonding mechanisms between (typically male) friendship or peer groups, within schools, clubs, or teams (Sales, 2016, 2017). Explanations for such bonding may lie in Male Peer Support Theory (DeKeseredy and Schwartz, 1993, 2016) which posits that in both offline and online contexts, violent men are likely to align with those peers who explicitly and implicitly support their misogynistic or violent tendencies. Indeed, DeKeseredy and Schwartz (2016) note that online behaviors facilitate wider displays of aggressive or violent masculinity as a form of peer recognition or bonding. Posting and viewing such images could also represent a way for men to assert heterosexual masculinity (Anderson, 2005; Waldron, 2015).

However, the only empirical study regarding OIBEV to our knowledge (Maas et al., 2021), found that 30% of women also viewed “slutpages,” albeit less frequently than men (40%). Given that most OIBEVS almost exclusively depict women, and are predominantly designed for the male gaze, this gives rise to questions about why women would view such sites. Potential explanations, derived from pornography research (Maas and Dewey, 2018; Davis et al., 2020) include curiosity or sexual arousal (including same-gender orientations), as well as wanting to know whether they themselves or their friends are depicted on these sites. However, specific motivations for OIBEV have not yet been empirically explored. Thus, these motivations are worthy of investigation, as understanding why people engage with or purposefully witness different forms of image-based sexual abuse can help inform prevention, policy development, and intervention programs (Duncan and March, 2019).

Uses and Gratification Theory (UGT) offers a framework to understand why individuals may seek out and use specific media to meet certain needs (Rubin, 2002) and has been applied to a range of online behaviors including cyberbullying (Hu, 2016; Tanrikulu and Erdur-Baker, 2019). Equally, it can be applied to OIBEV, which may serve a need to gratify via content (i.e., the images themselves) or serve process gratification needs (i.e., purposefully navigating to answer a question) by exploring whether one’s images have been posted. To test this theory, we proposed to explore expressed motivations for OIBEV. Additionally, we were interested in analyzing associations with other forms of image-based sexual abuse, such as non-consensual sext dissemination, and technology-facilitated violence, such as cyberbullying, given conceptual similarities. By extension, OIBEV victimization (and visiting sites to see if one is depicted) might overlap with sext dissemination victimization and cyberbullying victimization. Further, visiting OIBEV sites to view/check out images might be associated with engagement in other harmful forms of technology-facilitated violence, such as sext dissemination and cyber-bullying perpetration.

Sext dissemination refers to the distribution of sexts (sexually explicit messages, images, or photos sent to others via electronic means) to people beyond the original intended audience, and often (although not exclusively) against the wishes or without the knowledge of the person depicted (Strasburger et al., 2019; Clancy et al., 2020). This can include posting images on OIBEV sites, as well as more private forms of dissemination via mobile phones. Victims of non-consensual dissemination may experience distress, anxiety and reduced self-esteem (Walker and Sleath, 2017), loss of employment (Citron and Franks, 2014; Cannon, 2015) and even post-traumatic stress symptoms and suicidal ideation (Bates, 2016). However, most of those engaging in sext dissemination report relatively innocuous motivations, such as thinking the individual was “hot,” as a joke or to be funny, or to enhance social status (Clancy et al., 2019, 2020), consistent with UGT that this behavior serves a functional purpose. Given the potential impact on victim mental health, and the potential for polyperpetration and/or polyvictimization, relationships between dissemination and OIBEV will also be explored.

Cyberbullying constitutes a form of online aggression, involving intentional and repeated harm inflicted on another via electronic means (Patchin and Hinduja, 2006). Perpetrators of cyberbullying derive gratification from their actions, perceiving themselves in a position of power relative to their victim(s; Patchin and Hinduja, 2006), and perpetration has been explained with reference to UGT, with cyberbullying offering opportunities for entertainment, revenge or domination of others (Hu, 2016; Tanrikulu and Erdur-Baker, 2019). Cyberbullying behaviors typically include threats, spread of harmful or untrue information about the targeted person(s), exclusion, and “outing,” acts of public sharing of private, intimate, and potentially embarrassing information or images of the victim(s; Willard, 2007). Both victims and perpetrators suffer adverse mental health outcomes including peer and emotional problems, psychiatric symptoms/disorders, lower self-esteem, substance use and conduct problems (Kowalski et al., 2014; Fisher et al., 2016; John et al., 2018).

Parallels between OIBEV, non-consensual dissemination and cyberbullying are striking. Those who post material to OIBEV sites are typically engaging in a form of non-consensual dissemination, with no consent obtained for the sharing of such images, and become cyberbullying perpetrators as the intention behind this online behavior is to cause harm to the individual/s depicted. All three behaviors may serve functional outcomes, with positive experiences being reinforcing as they fulfill gratification needs as suggested by UGT. Analogously, being featured on OIBEV sites echoes cyberbullying and non-consensual dissemination victimization, with consequent experiences of significant distress and fear. Visiting sites, whether OIBEV sites or social media, to determine whether one is a victim and the extent of impact, would serve process gratification needs, consistent with UGT. As such, investigation of potential overlaps between “slutpage” use, sext dissemination perpetration and victimization, and cyberbullying perpetration and victimization, is of interest to see where prevention efforts could be mutually supportive. In addition, we aimed to investigate potential associations with depression, anxiety and stress for OIBEV site visitation, given previous proposed associations (Sales, 2016, 2017), and whether this differs with OIBEV site visitation motivations.

Lastly, given the paucity of information regarding OIBEV site visitation generally, it is of value to document this behavior in two nations, investigate potential cross-national differences, and determine any trends in rates of behaviors between different nations which can help explain these behaviors. This will be of interest to those seeking to develop programs that can prevent such behaviors, particularly those developing school-based sexual and relationship health education programs. In addition, this information will inform service responses for victim-survivors of this form of image-based sexual abuse.

Recent research exploring non-consensual sexting behaviors across various nations has found striking differences. In a study involving participants from ten nations across four continents (Morelli et al., 2020), there was considerable variation in rates of non-consensual dissemination based on nationality and personality traits. However, the overall model explained only 5% of variance, hence such differences may possibly be explained by factors other than personality traits and nationality.

One potential explanation could be the difference in school-based sexual health and relationships education programs, which vary between nations in their reference to online behaviors. Comparing Australian and United States programs provides an illustration. Australian schools are mandated to provide comprehensive sexuality education as part of school curricula, addressing physical sexual health, social issues of relationships and consent (Smith et al., 2011). More recent materials are increasingly focusing on online as well as offline behaviors (Fisher et al., 2019). By contrast, United States requirements show significant variation between states, with some states emphasizing abstinence approaches as the preferred approach for preventing pregnancy and sexually transmitted infections, limiting information about safe sex practices (Peters, 2007; Ford et al., 2013), whilst broader discussions of sexual practices remain controversial (Mullinax et al., 2017). Differences in the use of OIBEV sites between United States and Australian participants may relate to differences in a range of factors, including differences in compulsory relationship and sexuality education.

Despite its presence in the popular press, the phenomenon of “slutpages” has received little empirical attention. As noted, only one study to date has assessed the prevalence of “slutpage” use and associated behaviors, within a United States college sample. To further build on this line of work, we aim to (1) identify the motivations for visiting OIBEV sites in the United States and Australia, and whether visitation frequency and ascribed motivations vary cross-nationally or by gender; (2) examine OIBEV site visitation with associated online behaviors, specifically sext dissemination and cyberbullying perpetration, and (3), identify associations with mental health. Drawing on available prior research, we proposed the following hypotheses:

1. Men would be more likely to visit OIBEV sites overall.
2. Men would be more likely to visit OIBEV sites to view images than women.
3. Women would be more likely to be motivated to visit OIBEV sites to see if they were depicted themselves than men.
4. OIBEV site visitation would be positively associated with sext dissemination and cyberbullying perpetration.
5. OIBEV site visitation to see if one was depicted would be positively associated with mental health concerns, specifically depression, anxiety, and stress, whereas there would be no association with negative mental health outcomes for frequenting OIBEV sites for general viewing.

Given minimal available prior research, no *a priori* hypotheses were generated regarding cross-national differences.

## Materials and Methods

### Participants

Participants in this study included 1148 young adults aged 18 to 29 years (*M* = 22.54, *SD* = 2.50); 53.0% women, and 47.0% men. Participants were residents of the United States (53.8%) and Australia (46.2%). The majority (80.7%) reported their sexual orientation as heterosexual, 14.5% as bisexual, 3.7% lesbian or gay, and 1.0% were unwilling to disclose their sexuality. Most (81.8%) reported being currently sexually active, with 17.19 years (*SD* = 2.44) being the average age of first sexual activity.

### Materials

#### OIBEV Site Visitation

Drawing on Maas et al. (2021), we determined whether participants had ever visited an OIBEV site with the following item: “Have you ever visited a secret page on social media (such as Instagram, Facebook, or Snapchat, etc.) or a website of nude images or video that were posted without the knowledge of those in the images/videos (sometimes referred to as Slutpages/Listservs)?”. Participants could respond with yes/no responses. Those responding in the affirmative were asked “What was your purpose?” and could respond with one of the following: *To check out the people on the page; To see if I was listed or depicted on the page* or *Other (please specify)*, with qualitative responses manually coded to identify themes.

#### Sext Dissemination Behaviors

This study adapted a previous sext dissemination questionnaire to focus on specific aspects of sexting. For the purposes of this study, all sexting-related questions referred to sexts as “sexually explicit images, sent, received or shared via mobile phone messaging or apps.” Sexting behaviors were assessed via questions about engagement in requesting, receiving, sending, and disseminating sexually explicit images via mobile phone messaging or apps, as well as frequency of dissemination (for all survey items, see Clancy et al., 2021). Participants were asked about both disseminating and receiving disseminated sexts; “Has someone ever forwarded you an image-based sext via text or mobile app that was not originally intended for you?” (*Yes/No*) and “Have you ever received an image-based sext intended for yourself which you subsequently showed/sent to another person?” (*Yes/No*). Participants were also asked whether they knew of their own images having been shared with others; “Have you ever sent an image-based sext of yourself that was subsequently forwarded (to your knowledge?).” If they were aware of this occurring, they were also asked whether they had consented; “Had you given permission for this image to be forwarded?” with responses options of *Yes/No*.

#### Cyberbullying Perpetration and Victimization

Cyberbullying victimization and perpetration were assessed using measures drawn from Ybarra et al. (2007). Each scale consist of three items, addressing how frequently in the past year the individual engaged in cyberbullying behaviors, or was the target of such behaviors. A sample item is: “In the last year, how many times did you receive rude or nasty comments from someone while online?” Response options provided were *everyday/almost every day, once or twice a week, once or twice a month, a few times a year, less than a few times a year*, and *never*. Participants who reported any of the three experiences in the previous year were coded as having experienced cyberbullying/perpetrated cyberbullying. Responses were reverse coded for analysis, so that higher scores on the scale indicate a greater degree of cyberbullying behaviors. These scales had good reliability, with ordinal alpha scores of 0.83 and 0.88 for victimization and perpetration, respectively.

#### Depression, Anxiety, and Stress

Depression, anxiety and stress symptoms were measured using the short form of the Depression, Anxiety and Stress Scale (DASS-21; Lovibond and Lovibond, 1995). This 21-item self-report instruments includes three 7-item subscales, and participants indicate how much this statement has applied to them over the past week, using a 4-point Likert scale ranging from 0 (*did not apply to me at all*) to 3 (*applied to me very much, or most of the time*). Responses for each subscale are summed, and higher scores indicating greater levels of psychological distress. The DASS-21 has demonstrated good psychometric properties (Henry and Crawford, 2005) and internal reliability in this sample was good, with Cronbach’s alphas for depression, anxiety and stress subscales of 0.93, 0.87, and 0.89, respectively.

### Procedure

After obtaining ethics approval from the Deakin University Human Research Ethics Committee, participants were recruited through social media (Facebook, Instagram, and Reddit), as well as survey aggregator sites Amazon MTurk (*N* = 702) and Prolific (*N* = 136), which were used to achieve balanced samples by gender and country. Planned contrasts between social media and survey aggregator participants identified that those recruited via social media were more likely to be women (*t* = 20.26, *p* < 0.001), and resident in Australia (*t* = –34.51, *p* < 0.001), whilst there was no significant difference in age (*t* = –1.93, *p* = 0.06). Social media participants were more likely to have disseminated sexts than those from survey aggregator sites (*t* = 2.24, *p* = 0.026), although the absolute value of this difference was small (Facebook 15%, survey aggregators 13%). Social media participants were also more likely to report either cyberbullying perpetration (*t* = 5.08, *p* < 0.001) or victimization (*t* = 2.62, *p* = 0.009), although again absolute differences were small (perpetration: Facebook *M* = 16.97, survey aggregators *M* = 16.01; victimization: Facebook *M* = 15.33, survey aggregators *M* = 14.56). Social media participants were less likely to have visited OIBEV sites (*t* = –3.50, *p* = 0.001; Facebook 14%, survey aggregators 22%).

Across all sites, advertisements informed participants that the study aimed to explore factors that can influence sexting behaviors, was open to adults aged 18–29 years, regardless of whether they had sexted or not, and emphasized response anonymity. Potential participants reviewed a brief online study description and indicated consent by commencing the survey, which took 15–20 min to complete. Participation was voluntary and confidential, with no incentive offered for general social media participants. MTurk participants received a payment of $1USD per survey completion, whilst Prolific participants received a payment of £1GBP. Survey responses were gathered during July-October 2019.

### Analyses

All analyses were conducted using SPSS V26. Descriptive statistical analyses were used to review sample and variable characteristics. For all inferential analyses, an error level of 5% was used to determine results as significant. Analysis of difference by gender was conducted using chi-squared analyses, and point-biserial correlations between independent variables and sext dissemination were conducted. Binomial multivariate logistical regressions were conducted to understand the impact of behavioral and personality variables on sext distribution. Multivariate regression analyses were used to identify the unique and combined contribution of all variables in explaining variance in OIBEV site visitation. As the same data set was used for all analyses, Bonferroni corrections were employed to adjust for inflated risk of Type 1 errors. A *post hoc* sensitivity analysis using GPower (Erdfelder et al., 1996) identified a minimum required sample size of 761, with the current sample exceeding this (at *N* = 1148). Inductive coding of qualitative responses was conducted by the first author (EC), with a random sample of responses (10%) reviewed by the last author (BK). No discrepancies were noted in this analysis.

## Results

Descriptive statistics for key variables are provided in Table 1. OIBEV site visitation was significantly more common in the United States sample (23%) than the Australian sample (16%), χ (1) = 7.93, *p* = 0.005, Fisher’s *Z*_*r*_ = 0.19, indicating a small effect size. Testing Hypothesis 1, we found that rates of visiting OIBEV sites for men were almost double those for women overall, and this was similar across both the Australian and United States samples, with moderate effect sizes. The most common reason provided for visiting these sites was to “check it out,” but this varied depending on gender. Confirming Hypothesis 2, men were most likely (80%) to be visiting the site to “check it out.” Women were equally likely to be visiting to “check it out” (41%) and to see if they were depicted on the site (36%). To see if they were depicted was chosen notably more for women than men and is consistent with Hypothesis 3. Participants had the option to record “other” reasons for OIBEV site visitation and provide a free-text reason if desired. Of participants who had visited OIBEV sites, 36 (16%) selected this, with 30 providing text responses, including 16 men and 20 women. These responses were inductively coded to arrive at themes, with multiple themes noted for some responses. Additional reasons included curiosity (*n* = 12, 42% men), with indicative comments including “*I was a curious teenager and saw that people were looking at these pages: I had a look and decided it was wrong,”* and *“stumbled upon it and curiosity got me, just looking at what was on there.”* The other relatively common reason was accidental visits (*n* = 9, 67% men), e.g., “*I came across the page/s unintentionally, several times across several years,” “Given a link, didn’t know where it led.*” Six participants (all women) noted helping a friend (*n* = 6, e.g., “*My friend was on the page and I had to go on and report photos*”), and self-gratification was also noted by three participants (all men) “*For pornographic gratification.*”

**TABLE 1:** Descriptive statistics and chi square comparisons by gender for OIBEV site visitation and visitation reasons.

TOTAL (*N* = 1148)	% Men (*n* = 540)	% Women (*n* = 608)	Total	Chi-square test
Ever visited OIBEV site (% Y)	26% (139)	14% (86)	20% (225)	χ^2^ (1) = 24.41, *p* < 0.001, Fisher’s *Z*_*r*_ = 0.34
Visitation reasons: To check it out (% of OIBEV site visitors)	80% (110)	41% (35)	65% (145)	χ2 (2) = 37.59, *p* < 0.001, Cramer’s *V* = 0.41
To see if I was on it	9% (12)	36% (31)	19% (43)	
Other*	12% (16)	23% (20)	16% (36)	

**Australia (*N* = 530)**	**% Men (*n* = 233)**	**% Women (*n* = 297)**	**Total**	**Chi-square test**

Ever visited OIBEV site (% Y)	24% (55)	10% (30)	16% (85)	χ^2^ (1) = 17.68, *p* < 0.001, Fisher’s *Z*_*r*_ = 0.49
Visitation reasons: To check it out (% of OIBEV site visitors)	70% (38)	30% (9)	56% (47)	χ2 (2) = 16.84, *p* < 0.001, Cramer’s *V* = 0.45
To see if I was on it	7% (4)	40% (12)	19% (16)	
Other*	22% (12)	30% (9)	25% (21)	

**United States (*N* = 618)**	**% Men (*n* = 307)**	**% Women (*n* = 311)**	**Total**	**Chi-square test**

Ever visited OIBEV site (% Y)	27% (84)	18% (56)	23% (140)	χ^2^ (1) = 7.72, *p* = 0.005, Fisher’s *Z*_*r*_ = 0.24
Visitation reasons: To check it out (% of OIBEV site visitors)	86% (72)	46% (26)	70% (98)	χ^2^ (2) = 24.73, *p* < 0.001, Cramer’s *V* = 0.42
To see if I was on it	10% (8)	34% (19)	19% (27)	
Other*	5% (4)	20% (11)	11% (15)	

* respondents could provide additional information, which was manually coded.

To test Hypothesis 4, we ran point-biserial correlations to determine whether OIBEV site usage was associated with other key variables of interest including sexting behaviors, cyberbullying and mental health indicators, as presented in Tables 2 and 3. Given noted gender disparities in prevalence and viewing reasons, we conducted analyses using the overall sample and split by gender. Visiting OIBEV sites was significantly associated with gender (men more likely), and positively associated with sext dissemination perpetration and victimization, requesting sexts and receiving disseminated sexts, providing partial support for Hypothesis 4. However, OIBEV site visitation was negatively associated with both cyberbullying victimization and perpetration, in contrast to our hypotheses. When considering mental health, OIBEV site visitation was positively associated with anxiety and depression. However, all significant associations were weak, with the strongest relationship between OIBEV site visitation and dissemination receipt explaining only 4% of variance.

**TABLE 2:** Point-biserial Correlations between OIBEV site visitation, online behaviors and mental health: full sample.

	Gender	Age	Sexually	Received	Sent	Dissem.	Requested	Disseminated	Dissem.	Anxiety	Depression	Stress	CBV	CBP
			active	sext	sext	victim			receipt					
OIBEV site visitation	−0.15**	0.03	–0.04	0.01	0.01	0.14**	0.12**	0.15**	0.19**	0.07*	0.08*	0.04	−0.11**	−0.18**
Gender		−0.09**	0.15**	0.11**	0.15**	0.08**	−0.16**	–0.02	–0.04	0.08*	< −0.01	0.14**	0.10**	0.15**
Age			0.10**	0.06*	–0.03	–0.03	0.11**	0.06	–0.05	−0.09**	–0.06	–0.06	–0.01	–0.04
Sexually active				0.37**	0.29**	0.12**	0.18**	0.07*	0.06	0.03	−0.07*	–0.02	<0.01	0.01
Received sext					0.51**	0.14**	0.35**	0.16**	0.14**	0.10**	0.06	0.08*	−0.10**	–0.05
Sent sext						0.22**	0.49**	0.13**	0.12**	0.01	< −0.01	0.07*	0.01	0.08*
Diss. victim							0.17**	0.25**	0.22**	0.11**	0.04	0.09*	−0.13**	−0.08*
Requested								0.18**	0.12**	<0.01	0.01	0.02	–0.06	−0.07*
Dissemination									0.18**	0.10**	0.04	0.10**	−0.16**	−0.22**
Diss. receipt										0.10**	0.06	0.07*	−0.09**	−0.09**
Anxiety											0.71**	0.77**	−0.28**	−0.18**
Depression												0.76**	−0.23**	−0.14**
Stress													−0.20**	−0.11**
CBV														0.69**
CBP														

Dissem, Dissemination; CBV, Cyberbullying Victimization; and CBP, Cyberbullying perpetration. * = <0.05, ** = <0.01.

**TABLE 3:** Point-biserial Correlations of OIBEV site visitation, online behaviors and mental health by gender: Upper triangle Men, lower triangle Women.

	OIBEV	Country	Age	Sexually	Received	Sent	Victim	Requested	Diseminateds	Dissem	Anxiety	Depression	Stress	CBV	CBP
	visitation			active	sext	sext	Victim			receipt					
OIBEV site visitation		0.04	0.03	–0.03	0.01	0.03	0.13**	0.09*	0.21**	0.21**	0.09	0.13**	0.13**	−0.09*	−0.17**
Country	0.11**		0.28**	0.10*	0.13**	0.10*	–0.03	0.09*	0.09*	−0.14**	0.05	–0.02	–0.08	−0.14**	−0.15**
Age	0.01	0.42**		0.24**	0.22**	0.09*	0.10*	0.15**	0.11**	–0.02	0.00	–0.02	–0.01	–0.03	–0.04
Sexually active	0.00	–0.01	–0.03		0.41**	0.24**	0.13**	0.24**	0.12**	0.06	0.01	–0.08	–0.06	–0.03	–0.05
Received sext	0.04	–0.03	−0.09*	0.30**		0.52**	0.13**	0.47**	0.14**	0.18**	0.08	0.03	0.03	−0.10*	–0.08
Sent sext	0.04	–0.05	−0.11**	0.31**	0.49**		0.20**	0.62**	0.17**	0.20**	–0.04	–0.04	–0.02	–0.06	0.01
Dissemination victim	0.19**	–0.03	−0.11**	0.09*	0.14**	0.23**		0.16**	0.24**	0.24**	0.09*	0.04	0.11*	–0.07	–0.08
Requested	0.10*	0.10*	0.05	0.18**	0.26**	0.43**	0.21**		0.24**	0.18**	–0.01	–0.02	0.01	–0.06	–0.07
Dissemination	0.09*	–0.02	0.00	0.02	0.10*	0.11**	0.27**	0.13**		0.26**	0.03	–0.04	0.02	−0.14**	−0.24**
Dissemination receipt	0.17**	−0.11**	−0.08*	0.07	0.10*	0.06	0.21**	0.06	0.10*		0.07	0.03	0.06	–0.07	–0.06
Anxiety	0.09	–0.08	−0.18**	0.03	0.09	0.04	0.12*	0.04	0.16**	0.13**		0.73**	0.78**	−0.31**	−0.27**
Depression	0.01	0.00	−0.11*	–0.05	0.10*	0.03	0.04	0.04	0.13**	0.10*	0.69**		0.77**	−0.23**	−0.16**
Stress	–0.02	–0.09	−0.12*	–0.02	0.11*	0.12*	0.05	0.07	0.19**	0.08	0.76**	0.76**		−0.24**	−0.19**
CBV	−0.10*	−0.10*	0.02	0.00	−0.12**	0.06	−0.19**	–0.03	−0.18**	−0.11*	−0.26**	−0.23**	−0.19**		0.70**
CBP	−0.14**	−0.13**	–0.02	0.05	–0.05	0.12**	−0.11*	–0.02	−0.19**	−0.11*	−0.11*	−0.12*	–0.07	0.66**	

Dissem, Dissemination; CBV, Cyberbullying Victimization; and CBP, Cyberbullying perpetration. * = <0.05, ** = <0.01.

When split by gender, similar patterns were noted, but with some important differences (see Table 3). For men, both dissemination perpetration and receipt of disseminated images were most closely associated with OIBEV site visitation, whilst dissemination victimization and requesting sexts were less strongly, although still significantly associated with OIBEV site visitation. Both cyberbullying victimization and perpetration were negatively associated with visiting OIBEV sites, depression and stress were positively associated with visitation, but anxiety was not associated. For women, visiting OIBEV sites was associated with being from the United States rather than Australia, and most strongly and positively associated with dissemination victimization and receipt of disseminated sexts. There were no associations between OIBEV site visitation and mental health variables for women. Given the relatively low frequency of such behaviors, correlational analyses were not split by motivations for OIBEV site visitation.

To determine the most impactful factors associated with visiting OIBEV sites, the sexting, mental health and cyberbullying variables, as well as demographics (gender, age, and country) were entered into a hierarchical regression to predict OIBEV site visitation (see Table 4). The overall model was significant; [χ^2^ (15) = 93.90, *p* < 0.001], predicting 17% of variance (Nagelkerke’s *R*-square). Unique factors predicting OIBEV site visitation included gender (β = 0.58, *p* = 0.007), with women half as likely as men to visit OIBEV sites, and country (β = 1.76, *p* = 0.011), Americans more likely than Australians to visit these sites. Considering behavioral associations, dissemination victimization (β = 2.25, *p* = 0.002), having requested sexts (β = 1.18, *p* = 0.012) and receipt of disseminated images (β = 2.14, *p* < 0.001) were associated with increased likelihood of having visited an OIBEV site, whereas cyber-bullying perpetration (β = 0.90, *p* = 0.009) was associated with reduced odds of OIBEV site visitation.

**TABLE 4:** Hierarchical regression results – predictors of OIBEV site visitation overall.

	*Chi square*	*df*	*P*	*–2 Log likelihood*	Nagelkerke *R*-squared	
Step 1	21.44	3	<0.001	802.78	0.04	
Step 2 - change	58.77	7	<0.001	744.01	0.15	
Step 3 – change	3.45	3	0.327	740.55	0.15	
Step 4 - change	9.23	2	0.010	731.32	0.17	
Overall model	93.90	15	<0.001	731.32	0.17	

	***B***	***Wald***	***p***	**OR**	**95% CI for OR**	
					**Lower**	**Upper**

Gender^1^	–0.55	7.30	0.007	0.58	0.39	0.86
Country^2^	0.56	6.53	0.011	1.76	1.14	2.71
Age	–0.01	0.10	0.755	0.99	0.91	1.07
Sexually active	–0.45	2.91	0.088	0.64	0.78	1.07
Received sexts	–0.13	0.14	0.711	0.88	0.45	1.72
Sent sexts	–0.16	0.38	0.539	0.84	0.51	1.42
Dissemination victim^3^	0.81	9.24	0.002	2.25	1.33	3.81
Requested sext^3^	0.60	6.33	0.012	1.82	1.14	2.91
Dissemination receipt^3^	0.76	14.18	<0.001	2.14	1.44	3.18
Dissemination^3^	0.38	2.17	0.141	1.46	0.88	2.42
Anxiety	0.01	0.01	0.952	1.00	0.97	1.03
Depression	0.02	1.37	0.242	1.02	0.99	1.04
Stress	–0.01	0.14	0.706	0.99	0.96	1.03
CBV	0.02	0.29	0.591	1.02	0.95	1.10
CBP	–0.10	6.83	0.009	0.90	0.83	0.97
Constant	0.18	0.02	0.879	1.20		

*Note – coefficients for individual predictors only provided for Step 4 (final solution). Coefficients at earlier steps available from authors.*

*^1^Gender coded Men = 1, Women = 2.*

*^2^Country coded Australia = 0, United States = 1.*

*^3^All other binary variables coded 0 = No, 1 = Yes.*

When analyzing men and women separately, both models were significant. For men, the overall model predicted 19% of variance (Table 5), χ^2^ (14) = 58.35, *p* < 0.001. Unique factors which predicted male OIBEV site visitation included having requested sexts (β = 2.17, *p* = 0.020), disseminated sexts (β = 2.35, *p* = 0.012), receipt of disseminated images: (β = 1.99, *p* = 0.012), lower levels of anxiety: (β = 0.92, *p* = 0.028), and lower likelihood of cyberbullying perpetration: (β = 0.90, *p* = 0.042). For women, the model predicted 17% of variance (Table 6), χ^2^ (14) = 41.26, *p* < 0.001. Unique factors which predicted female OIBEV site visitation included country: (β = 1.56, *p* = 0.025), with American women more likely than Australian women to visit these sites; sext dissemination victimization (β = 2.53, *p* = 0.017), receipt of disseminated images (β = 2.42, *p* = 0.006), higher levels of anxiety (β = 1.07, *p* = 0.009) but reduced stress (β = 0.94, *p* = 0.032).

**TABLE 5:** Regression results – predictors of OIBEV site visitation for men.

	*Chi square*	*df*	*P*	*–2 Log likelihood (change)*	Nagelkerke *R*-squared	
Step 1	1.166	2	0.435	495.39	0.01	
Step 2 - change	29.62	7	<0.001	434.83	0.14	
Step 3 – change	9.62	3	0.022	425.21	0.17	
Step 4 - change	7.44	2	0.024	417.77	0.19	
Overall model	58.35	14	<0.001	417.77	0.19	

	***B***	**Wald**	***p***	**OR**	**95% CI for OR**	
					**Lower**	**Lower**

Country^1^	0.24	2.73	0.099	14.27	0.96	1.68
Age	–0.02	0.12	0.727	0.98	0.88	1.09
Sexually active	–0.53	2.77	0.096	0.58	0.31	1.10
Received sexts	0.07	0.03	0.872	1.07	0.46	2.48
Send sexts	–0.45	1.83	0.176	0.64	0.33	1.23
Dissemination victim^2^	0.63	2.67	0.102	1.88	0.88	4.02
Requested sext^2^	0.77	5.41	0.020	2.17	1.13	4.15
Dissemination receipt^2^	0.69	6.27	0.012	1.99	1.16	3.40
Dissemination^2^	0.85	6224	0.012	2.35	1.20	4.59
Anxiety	–0.05	4.84	0.028	0.92	0.90	0.99
Depression	0.03	2.59	0.107	1.03	0.99	1.06
Stress	0.03	2.23	0.135	1.03	0.99	1.08
CBV	0.00	0.00	0.964	1.00	0.90	1.11
CBP	–0.11	4.14	0.042	0.90	0.81	0.99
Constant	–0.33	0.05	0.829	0.72		

*^1^Country coded Australia = 0, United States = 1.*

*^2^All other binary variables coded 0 = No, 1 = Yes.*

**TABLE 6:** Regression results – predictors of visiting OIBEV site visitation for women.

	*Chi square*	*df*	*P*	*–2 Log likelihood*	Nagelkerke R-squared	
Step 1	6.20	2	0.045	326.51	0.03	
Step 2 – change	24.20	7	0.001	302.31	0.13	
Step 3 – change	8.87	3	0.031	293.44	0.17	
Step 4 - change	1.99	2	0.370	291.45	0.17	
Overall model	41.26	14	<0.001	291.45	0.17	

	***B***	**Wald**	***p***	**OR**	**95% CI for OR**	
					**Lower**	**Lower**

Country^1^	0.44	5.02	0.025	1.56	1.06	2.29
Age	–0.01	0.02	0.885	0.99	0.86	1.14
Sexually active	–0.35	0.38	0.534	0.70	0.23	2.13
Received sexts	–0.26	0.17	0.678	0.77	0.23	2.61
Send sexts	0.34	0.50	0.480	1.40	0.55	3.59
Dissemination victim^2^	0.93	5.68	0.017	2.53	1.18	5.44
Requested sext^2^	0.31	0.77	0.380	1.36	0.69	2.69
Dissemination receipt^2^	0.88	7.42	0.006	2.42	1.28	4.57
Dissemination^2^	–0.14	0.10	0.757	0.57	0.35	2.15
Anxiety	0.06	6.86	0.009	1.07	1.02	1.12
Depression	0.01	0.04	0.836	1.01	0.96	1.05
Stress	–0.06	4.60	0.032	0.94	0.89	0.99
CBV	0.02	0.06	0.81	1.02	0.90	1.14
CBP	–0.08	1.63	0.20	0.92	0.81	1.05
Constant	–1.75	0.71	0.401	0.17		

*^1^Country coded Australia = 0, United States = 1.*

*^2^All other binary variables coded 0 = No, 1 = Yes.*

A final binary logistic regression analysis was used to assess Hypothesis 5, and determine factors associated with the two main reasons for OIBEV site visitation: only those who had reported either having visited to “check it out” or “to see if they were depicted” were included in this analysis due to small numbers in other categories. Potential predictors in the model included age, gender, country, having requested, sent or received sexts, disseminating and receiving disseminated sexts, and mental health and cyberbullying variables. Overall, the model was significant; χ^2^ (13) = 46.02, *p* < 0.001, predicting 42% of variance. In contrast to our hypotheses, the only unique predictor was gender (β = 9.15, *p* < 0.001), with women nine times more likely to be viewing OIBEV sites to see if they were depicted, compared to men.

## Discussion

This study aimed to identify the motivations for OIBEV site visitation across United States and Australian samples, examine differences in ascribed motivations cross-nationally and by gender, examine associations between OIBEV site visitation and other online behaviors such as sexting and cyberbullying, and associations with mental health. We proposed that men would be more likely than women to visit OIBEV sites overall, and to visit OIBEV sites to view images, whilst women would be more likely than men to visit OIBEV sites to see if they were depicted themselves. We also hypothesized that OIBEV site visitation would be positively associated with sext dissemination and cyberbullying perpetration, and that visiting a site to see if one was depicted would be associated with depression, anxiety, and stress. No *a priori* hypotheses were generated regarding cross-national differences.

Overall, our hypotheses were largely supported. Men were twice as likely to visit OIBEV sites as women across both nations, supporting Hypothesis 1. Of those who did report OIBEV site visitation, men were twice as likely as women to have visited to “check it out,” whilst women were 3–4 times more likely than men to have visited to see if they were themselves depicted on the site, consistent with Hypotheses 2 and 3. These results also support prior journalistic reporting (Sales, 2016, 2017) that OIBEV sites largely depict women and are largely created for sharing and consumption amongst heterosexual men. For men, OIBEV may provide gratification needs as proposed by Rubin’s (2002) UGT and relate to gratification via sexual arousal or ego needs (Henry and Flynn, 2019), much like revenge pornography (Walker and Sleath, 2017). In contrast, women may be meeting gratification by visiting OIBEV sites to determine if they are depicted on those sites and appease their concerns.

Providing partial support for Hypothesis 4, OIBEV site visitation was positively associated with a range of sexting behaviors, particularly having requested sexts from others, receiving disseminated images, having disseminated images to others, having one’s own image disseminated and requesting sexts from others. These findings were consistent for both men and women. However, dissemination perpetration and receipt were stronger predictors for men, whilst dissemination victimization was the strongest multivariate predictor for women and not significant for men. These findings support gendered differences in motivations for OIBEV site visitation, whereby men are more likely to visit OIBEV sites to view those depicted. This supports propositions that these men may also engage in other forms of technology facilitated abuse within peer groups, such as sending and receiving disseminated images (Henry and Powell, 2015).

By contrast, women, who were more likely to visit OIBEV sites to see if they were depicted, were also more likely to have had their own images shared with others. Whilst there was insufficient power to test for this, it is noted from other studies that, of those who are aware that their intimate images have been disseminated, less than 10% of women had given consent for this (Clancy et al., 2020). Our results are consistent with previous suggestions (Garrity and Blinder, 2015; Sales, 2016, 2017) that many OIBEV site images are non-consensually obtained, as well as shared. Of note, these gendered findings applied across both United States and Australia samples, with no difference by country noted, suggesting similar mechanisms are at work in both nations.

Interestingly, OIBEV site visitation was negatively associated with cyberbullying perpetration, overall and individually for men, but not for women, contrary to Hypothesis 4. Although researchers and policy makers may consider a conceptual overlap, with OIBEV constituting a form of cyberbullying, our results indicate that these behaviors are not correlated in the view of respondents. In fact, participants were less likely to visit OIBEV sites if they had been either a perpetrator *or* a victim of cyberbullying in the 12 months prior. Perhaps motivations associated with cyberbullying, such as harm, revenge, dominance, and entertainment (Tanrikulu and Erdur-Baker, 2019) differ (at least in part) from the motivations for OIBEV site visitation.

Considering the potential associations between OIBEV site visitation and mental health indicators (Hypothesis 5), we found that for men, OIBEV site visitation was associated with reduced anxiety, although effect sizes were small. For women, there was a small association between increased anxiety, but reduced stress, and OIBEV site visitation. These findings are unexpected, given previous journalistic reports regarding OIBEV victims (Sales, 2016, 2017) suggest significant mental health impacts, similar to those reported in association with other forms of technology facilitated sexual abuse (McGlynn et al., 2017; Powell et al., 2018). However, it is likely that lasting mental health impacts only occur for individuals if they are indeed depicted on a OIBEV site, and not simply investigating their own depiction. Anxiety about potential depiction for women may drive visitation, but reasons for our findings related to stress are unclear. However, these findings may also be indicative of the time elapsed since visiting OIBEV sites, with any distress having dissipated in the interim. Further investigation of the immediate impacts of OIBEV victimization, and confirmation of whether someone was depicted, would be useful additions for advancing understanding of this form of image-based sexual abuse and the impact on victims.

We also analyzed the sample to determine unique factors associated with OIBEV site visitation overall and by gender. Overall, men, people from the United States, those who had requested sexts themselves, received disseminated sexts, and had their own sext disseminated to others were more likely to visit OIBEV sites than women, people from Australia, or those who had never had their own sexts disseminated. However, those who identified as previous cyberbullying perpetrators were less likely to visit OIBEV sites than those who had never cyberbullied. These associations differed somewhat by participant gender. Men who had disseminated sexts, received disseminated sext images, and were lower in anxiety and cyberbullying perpetration were more likely to have visited OIBEV sites than those who had not engaged in these behaviors. For women, increased likelihood of having visited OIBEV sites was associated with being United States residents, having experienced sext dissemination victimization, receiving disseminated images themselves, higher levels of anxiety, but lower levels of stress.

These findings are consistent with prior findings that sext dissemination behaviors themselves are associated with normalization through peer groups, including exchanging (both sending and receiving) disseminated images (Clancy et al., 2019, 2020), and suggest at least some pathways by which images may be shared to then be posted on OIBEV sites. Of note, Clancy et al. (2020) found that disseminated sext images are usually received from someone of a different gender, but shared or circulated to those of the same gender, which may reinforce gender norms (DeKeseredy and Schwartz, 2016) of sharing such images in social groups, including via OIBEV sites.

The only unique predictor to distinguish between OIBEV site visitation to “check out” images and seeing if one was depicted on the site was gender, with women nine times more likely than men to have visited OIBEV sites to see if they were depicted. Whilst other site visitation motivations were relatively infrequent, limiting analytical power, helping out friends was only nominated by women, self-gratification was only endorsed by men, whilst both curiosity and accidental visits were relatively evenly distributed between men and women. These findings provide support for contentions that OIBEV site visitation, at least for the purposes of “checking out” the images, seems to reinforce problematic gender norms, in which women are depicted for the gratification of men (DeKeseredy and Schwartz, 2016). Further, our findings also support conceptualization of image-based sexual abuse, at least in this form, as a gendered behavior, perpetrated by men against women. We do note the potential for nuanced differences in more passive by standing and observing behaviors, as opposed to those who actively post images on these sites or rate those depicted, which was not specifically explored in this study. However, passive and voyeuristic viewing of images can still be seen as expanding the circle of those consuming the images, and as such, is still a form of image-based abuse, even if less active.

Considering intercultural differences, overall, United States respondents were more likely to have visited OIBEV sites than Australian participants, however, gender-based differences were more noticeable. In the hierarchical regressions, the combined demographic variables did not explain large amounts of variance, with sexting-related variables offering more explanatory power for OIBEV behaviors. Although our sample was not restricted to college or tertiary students, higher OIBEV frequencies in the United States may reflect higher engagement in tertiary residential social societies (e.g., sororities, fraternities) which are more common in United States institutions, and are associated with higher levels of OIBEV use (Maas et al., 2021).

Whilst study findings are novel and informative, some key limitations should be noted. In particular, this study was cross-sectional in design, and based on a convenience sample, and was not population representative for either nation, nor was it representative of broader cross-national groupings, all of which may limit generalizability of our findings. Further, participants were asked to self-report on their own behaviors which may impact accuracy. Whilst all efforts were made to ensure confidentiality of responses, and participants were non-identifiable, some may have been reticent to report engaging in behaviors which they deem at the least socially unacceptable, if not explicitly criminal. Additionally, many behaviors were measured at a dichotomous level, whilst our motivations for OIBEV visitation were relatively limited, and did not include specifically visiting sites to post images, consistent with the exploratory nature of this study. These factors are likely to reduce the power of our analyses. Lastly, some behaviors in this study were relatively infrequent, hence limiting power to investigation of the two most prominent visitation reasons.

Further investigation of OIBEV site visitation is warranted. Such research should focus on larger and potentially representative samples. Further, future research may benefit from more nuanced questionnaires which identify a broader range of OIBEV site uses from more actively pernicious site creation and posting of images, rating and commenting behaviors, through viewing the images of others and checking if oneself or one’s friends are depicted. Such research will help to replicate and extend current results and inform prevention efforts.

This study has important implications for those engaged in developing and implementing programs to address forms of image-based sexual abuse. In particular, programs which seek to target and talk about cyberbullying and do not specifically delineate OIBEV as a form of cyberbullying may fail to address problematic OIBEV, as users do not seem to associate these behaviors themselves. Based on our findings, we recommend that image-based sexual abuse prevention programs focus on social and peer norms around the use of online images. Additionally, it is also recommended to explicitly reference OIBEV and “slutpages” as a form of image-based abuse in education and prevention programs targeting adolescents and young adults, given the potential exposure and harms which could be associated.

## Data Availability Statement

The raw data supporting the conclusions of this article will be made available by the authors, without undue reservation.

## Ethics Statement

The studies involving human participants were reviewed and approved by Human Research Ethics Committee Deakin University, Burwood, VIC, Australia. The patients/participants provided their written informed consent to participate in this study.

## Author Contributions

EC, BK, and MM: conceptualization and methodology. EC: formal analysis, data curation and analyses, and writing—original draft preparation. EC, BK, and DH: investigation and project administration. EC, BK, EM, MM, and DH: writing—review and editing. All authors contributed to the article and approved the submitted version.

## Conflict of Interest

The authors declare that the research was conducted in the absence of any commercial or financial relationships that could be construed as a potential conflict of interest.
